# Cost and budget impact analysis of a school-based vision screening programme in Cambodia and Ghana: Implications for policy and programme scale-up

**DOI:** 10.1016/j.hpopen.2021.100043

**Published:** 2021-06-24

**Authors:** Thomas Engels, Guillaume Trotignon, David Agyemang, Imran Khan, Kann Puthy, Liesbeth Roolvink, Elena Schmidt

**Affiliations:** a Sightsavers, 35 Perrymount Rd, Haywards Heath, RH16 3BW, UK; b Sightsavers Ghana Office, No. 58 Patrice Lumumba Rd., Accra, Ghana; c Primary Education Department, Ministry of Education Youth and Sport, Preah Norodom Blvd, Phnom Penh, Cambodia

**Keywords:** Vision screening, Refractive errors, Schoolchildren, School health and nutrition, Economic evaluation, Budget impact analysis, Costing, Low- and middle-income countries

## Abstract

Poor vision due to unaddressed refractive error in children is considered to be a public health problem in many low- and middle-income countries. Research shows that correcting refractive error with spectacles could have a positive impact on school attendance and academic performance for children. The aim of this study was to estimate the cost of integrating vision screening and provision of spectacles in existing school health programmes in Cambodia and Ghana. Budget impact analysis of the intervention scale up is also reported, including univariate and multivariate sensitivity analyses. This study suggests that the scale up of school-based vision screening programmes is affordable in resource limited settings, such as Cambodia and Ghana, considering the current education budgets, and providing there is sufficient in-country capacity to deliver such interventions at scale. The study highlights several policy and programme implications and provides suggestions for minimising costs and maximising efficiencies of vision screening in a school setting. Findings from this analysis can help education planners and international partners to improve their planning and budgeting processes for school-based interventions to improve health and learning outcomes for children in low- and middle-income countries.

## Highlights

• Scale up of the model school-based vision screening programmes is feasible in low-income settings.
• Study provides an incremental cost estimate for the implementation of school-based vision.
• School Health Integrated Programming project issued technical guidance for policy-makers and planners.
• Study identifies main cost drivers of a school-based vision programme.

## 1 Introduction

Around 1 billion people live with preventable or unaddressed vision impairment globally, including 123.7 million with unaddressed refractive error (URE) [1]. The burden disproportionally affects people living in low- and- middle-income countries (LMICs), underserved populations, and in rural communities.

URE and unaddressed presbyopia are the leading cause of moderate and severe visual impairment [1]. Refractive errors occur when there is a mismatch between the shape and length of the eye results in light focusing either in front or behind the retina causing blurred and/or distorted vision. URE can be easily diagnosed and treated with optical devices such as spectacles, contact lenses or refractive surgery. The global economic losses due to URE were estimated to be US$244 billion a year [2], [3]. The epidemiology of refractive error in children is complex as prevalence and types of refractive error vary within and between countries [1], [4], [5]. The World Health Organization (WHO) and the Lancet Global Health Commission on Global Eye Health (2021) estimate that around 19 to 22 million children live with visual impairment globally, 12 million are due to URE, with the highest prevalence in South Asia, Southeast Asia and Western Sub-Saharan Africa, potentially negatively affecting quality of life, education opportunities and livelihoods of millions of children [1], [6], [7]. Surveys conducted in a number of LMICs showed that URE in children is an important public health problem with the proportion of visual impairment in children caused by URE ranging from 55% in Chile to 93% in China [8].

Children with poor vision face multiple barriers in education, as it is commonly accepted that a significant part of learning during the first 12 years of life occurs through vision. Schoolchildren with URE are at a major disadvantage in a range of classroom activities [9], which affects their school attendance and academic performance [10], [11], [12], [13].

School Health and Nutrition (SHN) programmes use schools as platforms to deliver safe, simple, and effective interventions essential for child development and growth. These programmes provide an opportunity to address health needs (e.g. deworming provided by teachers, hearing screening etc.) and support the education goals of school age children by getting them into schools, keeping them in education, and improving their learning whilst there [14]. The third edition of the Disease Control Priorities Project (DCP3) recommends an essential package of interventions for child and adolescent health [15], which includes vision screening and correction of refractive error alongside deworming, insecticide-treated net promotion, key immunisations, oral health promotion, and school feeding with micronutrient fortification. The cost of vision screening and provision of ready-made spectacles in the essential package for LMICs is estimated at US$0.60 per child per year [16]. Evidence from mathematical simulation models also shows that annual vision screening in schools is a very cost-effective intervention. Although the cost-effectiveness ratios vary depending on the population size, URE prevalence and school enrolment rates, school-based vision programmes were shown to be cost-effective in all WHO regions with the cost per Disability Adjusted Life Year (DALY) averted ranging from I$67–130 in South-East Asia to I$ 458–734 in Europe [17], [18].

Whilst return on investment and cost-effectiveness are important criteria for policy-makers to decide on which intervention to implement, this information does not necessarily provide an assessment of the affordability, particularly in resource-poor settings. The scarcity of real-world and context specific data in LMICs often inhibits governments from making informed choices about public funding allocations or effective management of publicly funded services. The lack of primary data on the costs and affordability of school health and nutrition interventions is a barrier to mainstreaming these activities within education sector plans and inhibits efforts to improve the efficiency and sustainability of government-funded programmes.

The purpose of this cost and budget impact analysis (BIA) was to generate evidence for policymakers to assess the costs and affordability of including vision screening into SHN interventions and for the delivery of these programmes at scale. The study was undertaken as part of the School Health Integrated Programming (SHIP) project which was delivered in 2016 in four LMICs: Cambodia, Ethiopia, Ghana, and Senegal. The aim of the SHIP project was to test the feasibility of integrating vision screening and refractive error correction within SHN plans and to build countries’ capacities to implement these plans at scale. More specifically, this study calculates the incremental costs of integrated vision screening and considers the budget implications for programme scale-up at national level in two of these countries, Cambodia, and Ghana.

## 2 Methods

### 2.1 Study design

The SHIP project was managed by two international non-governmental organisations (NGOs), Sightsavers and Partnership for Child Development (PCD), with oversight from the World Bank and funding from the Global Partnership for Education. In Ghana, activities were implemented by Sightsavers and PCD, in collaboration with the Ministry of Health and the Ministry of Education. Whereas in Cambodia, the Ministry of Education and Youth managed the funds and directly implemented the activities with the support from The Fred Hollows Foundation, Sightsavers, and Krousar Thmey, a Cambodian organisation. Data from the SHIP projects in Cambodia and Ghana was collected retrospectively to measure the incremental cost of implementing school-based vision screening as part of an existing school health programme. A budget impact model was developed to estimate the budget required for scaling-up the programme nationally in all public primary and lower secondary schools in both countries. The budget impact analysis follows the guidelines from the International Society for Pharmacoeconomics and Outcomes Research (ISPOR) [19]. Both the cost and budget impact analysis were carried out using excel.

### 2.2 Description of the intervention

The project activities took place in-country between January and December 2016. The SHIP approach advocates for the delivery of basic screening by trained teachers using simple inexpensive screening kits and children are referred to eye care professionals when failing the vision screening test, or in the presence of any abnormal clinical signs or symptoms [20]. Near vision acuity is not specifically measured. However, near vision issues are screened (including hyperopia and binocularity) by asking children about symptoms of headaches and eye pain (related to near vision issues), as well as assessing the appearance of the eyes. A child with strabismus, amblyopia or near vision symptoms would be referred. All referred students are assessed by a mobile team of optometrists, and children who required further treatment were referred to the nearest eye care unit or facility. The mobile team is comprised of optometrists, who visit schools soon after the initial teacher screening. Ready-made spectacles are provided immediately after the refraction for children who need spectacles and who meet specific prescribing guidelines; for children with more complex prescriptions, custom-made spectacles are procured and delivered to the schools one to two weeks later. School-based vision screening by teachers have shown acceptable results in other contexts [19], [21], [22], [23], [24]. Other studies concluded that vision screening by teachers lack the required specificity and/or sensitivity, even if authors generally concede that better teacher training or strict screening process could improve these results [25], [26], [27].

### 2.3 Study settings

In Cambodia, SHIP was implemented in two adjacent districts, Banthey Srey and Angkor Thum, in Siem Reap Province. The districts have a joint population of 67,108 people [28]. A total of 126 teachers were trained and 12,440 primary schoolchildren were screened. Among them, 72 (0.6%) received spectacles and 22 (0.2%) were referred for other eye treatments. In Ghana, vision screening was implemented in Denkyembour district in the Eastern Region, which has a population of 78,841 people [29]. A total of 120 teachers were trained and 10,099 primary and junior high schoolchildren were screened. Of these, 78 (0.8%) received spectacles and 235 (2.3%) were referred for other treatments. Both countries followed a similar delivery model described in the SHIP technical guidelines that are publicly available [20]. The only key difference in terms of activity was that in Cambodia the approach was already supported by the Ministry of Education and Youth and there were already trainers of teachers available and who required no further training.

### 2.4 Data collection

Data was collected retrospectively between January and March 2018. Project output data were extracted from the SHIP project reports. Additional data such as population demographics and school statistics (enrolment rates, numbers of students, teachers and schools), were obtained from the national Education Management Information Systems (EMIS) and existing national surveys.

Expenditures were collected from project partner accounting systems and financial records. The study did not include costs incurred by project beneficiaries (i.e. children and their families), which, given the project settings, were minimal. The project’s budget included transportation allowances and the spectacles that were provided free of charge. The analysis was incremental and did not account for the costs of the available medical and school infrastructure and resources. Only expenditures related to the implementation of the programme in each country were considered. Also, the costs of transport to eye clinics for children requiring other treatments were covered by the programme and included in the analysis, but the costs of treatment itself were not (due to the lack of robust data).

### 2.5 Cost analysis

**Fig. 1:**
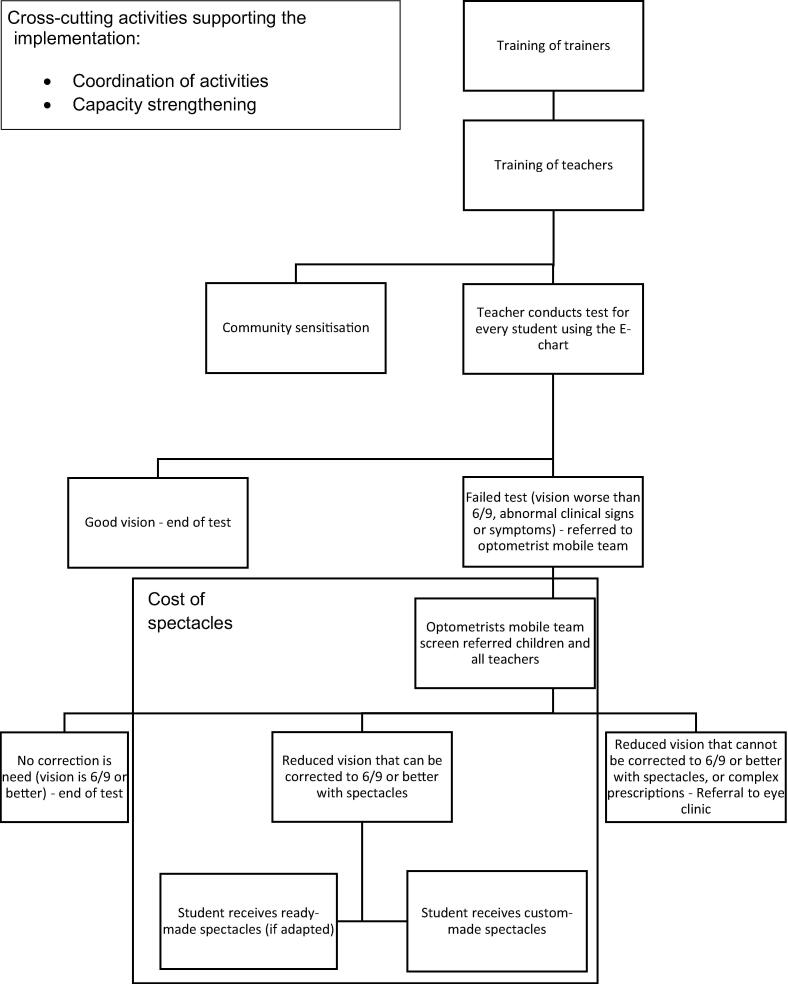
Diagram of school-health integrated programming standard activities included in the budget impact analysis.

The unit costs for standard activities as per guidelines adopted by the SHIP programme have been calculated (Fig. 1).

Although the activities in the two countries were closely aligned with the guidelines, there were some deviations reflected in the unit cost of each activity (see Table 1).

**Table 1:** Standard unit costs per school-based vision screening activities for Cambodia and Ghana; based on school-health integrated programming pilot data and technical guidelines (in USD 2020).

Activities	Unit	Cambodia	Ghana
		**Unit cost (USD)**	**Unit cost (USD)**
Capacity and partnership strengthening			
Workshop	Per participant per day	76	396
Training			
Training of trainers^a^	Per trainer	438	459
Training of teachers	Per teacher	93	72
Community sensitisation^b^			
Sensitization activity package	Per district	76	79
Sensitization activity package	Per region or province	218	210
Vision screening, eye examination, and treatment/referral			
Vision screening kit^c^	Per screening kit	3.9	4
Mobile refraction team^d^	Per day	315	255
Ready-made spectacle^e^	Per spectacles	2	2
Customized spectacle	Per spectacles	6.5	25
Transportation allowance for referral	Per child referred	20	21
Coordination, supervision and M&E^f^			
Coordination expenses at national level	Per year	10,296	38,400
Coordination expenses at sub-national level	Per year	13,310	28,738

Notes:

a No training of trainers were needed in Cambodia, costs were estimated based on the expenditure in Ghana, adjusted for local price level using Purchasing Power Parity (PPP) ratios.

b Cost for sensitisation includes radio messages, posters, and other activities for reaching out of school children and building awareness amongst school and community on the importance of vision screening and correcting refractive errors.

c One kit per school, screening kit for teachers contains a visual chart (6/9 optotype), a three meter rope and recording forms.

d Based on project expenditure and number of days worked by mobile refraction teams.

e Ready-to-clip spectacles used in SHIP were donated by Essilor; spectacles can be purchased for a unit price of 2 USD.

f Personnel involved in eye health programme as per SHIP guidelines: one programme manager and one technical manager (at central level), and one coordinator per administrative unit (either province or region).

For the activities that were recommended in the guidelines but not implemented in a given country (for example training of trainers in Cambodia), the standard costs were estimated based on the expenditure in the other country, adjusted for local price level using Purchasing Power Parity (PPP) ratios. Ready-made spectacles used in the SHIP project were donated. However, the value of donated spectacles was estimated using manufacturer prices and included in the standard costs.

The expenditures for the following activities have been included in the analysis: capacity and partnership strengthening (workshops); community sensitisation; coordination; training; screening at schools; treatment by mobile teams; spectacles; and referral to eye clinics (see appendix Table A). As we only consider incremental costs, salaries of teachers or optometrists have not been included as they would incur in the absence of the programme. However, per diems, transportation, and every other financial expenditure directly attributable to the programme have been incorporated in the cost analysis.

We used the average monthly market exchange rates for converting currencies, unless stated otherwise. All costs were then converted to 2020 US$ using inflation rates [30].

### 2.6 Budget impact analysis

The perspective adopted for the budget impact analysis is from the budget holder, that is the perspective of the government / Ministry of Education who will be taking over the implementation and scaling up of school-based vision screening in each country (Ministry of Education, Youth and Sport in Cambodia and the Ministry of Education in Ghana). Only the activities included in the standard SHIP package were considered for the BIA (Table 1). The costs of any international staff used in the SHIP project were adjusted using the costs of local personnel based on standard government per diems and salary grids.

The targeted population is all public primary school and lower secondary schools’ students and teachers of every provinces of Cambodia and every district of Ghana (Appendix Table C). Representing 94% and 72% of total primary school enrolment, and 98% and 85% of total secondary school enrolment respectively [31], [32]. The number of children enrolled were projected using a flow model and following the UNESCO / International Institute for Educational Planning (IIEP) technical guidelines [33]. Parameters such as promotion, repetition, and dropout rates were assumed to remain constant and equal to those observed in the base year (2016/17). Growth rates in new entrants were extrapolated from previous years (2011–2016) using both exponential growth rate and least square growth rate approaches [34]. A similar approach was used to estimate the number of new schools and teachers for each year.

The time horizon for the budget impact analysis is five years, which corresponds to the implementation of the Education Sector Strategy (2019–2023) in Cambodia and is aligned with the Education Strategic Plan in Ghana (2018–2030). A time horizon of one to five years is commonly used in BIA [19]. Prices were kept constant over the time horizon and no discounting was applied to future financial flows in the model.

All the key parameters and assumptions used in the budget impact model are outlined in the appendix (Table B). One-way and multi-way deterministic sensitivity analyses were performed to investigate uncertainties around certain parameters of the BIA model [35], [36]. For one-way sensitivity analysis, the value of one parameter was increased and decreased by 25% while holding other parameters constant, as seen in literature [37]. For the multi-way sensitivity analysis, the value of several selected parameters was simultaneously changed by 25% using all possible combinations.

### 2.7 Ethical considerations

The economic analysis was based exclusively on the review of secondary data and no primary data collection was required. Administrative approvals were obtained from the relevant Ministries in both countries prior to the study. All data management procedures ensured anonymity and confidentiality.

## 3 Results

### 3.1 Standard costs

Table 1 presents unit costs for a standard package in each country. The activities where unit costs were similar in Cambodia and Ghana were training of teachers; community sensitisation; screening kits; ready-made spectacles; and transportation for referrals. The unit costs that differed substantially were capacity strengthening ($76 per participant/day in Cambodia vs $396 in Ghana); mobile refraction teams ($315 per day in Cambodia and $255 in Ghana); custom-made spectacles ($6.5 per pair in Cambodia and $25 in Ghana); and coordination at the central ($10,296 per year in Cambodia and $38,400 in Ghana) and regional levels ($13,310 per province per year in Cambodia and $28,738 in Ghana).

### 3.2 Budget impact analysis

If the vision screening programmes are scaled up nationally, the overall resources required over five academic years (2018/19–2022/23) are projected to be $11,375,046 in Cambodia and $26,755,707 in Ghana (Table 2).

**Table 2:** Annual cost of national school-based vision screening programmes in Cambodia and Ghana, projection by activity for 2019–2023 (in USD 2020).

	**Cambodia**						**Ghana**					
	**Year 1**	**Year 2**	**Year 3**	**Year 4**	**Year 5**	**Total**	**Year 1**	**Year 2**	**Year 3**	**Year 4**	**Year 5**	**Total**
I**. Capacity and partnership strengthening**												
Number of participants (A1)	48					48	55					55
Cost per participants (A2)	81					81	428					428
Cost of national workshop (A)=(A1)x(A2)	3,910					3,910	23,519					23,519
**II. Community sensitisation**												
Number of region (B1)	25	25	25	25	25		10	10	10	10	10	
Cost per region (B2)	254	254	254	254	254		227	227	227	227	227	
Number of district (B3)	196	196	196	196	196		217	217	217	217	217	
Cost per district (B4)	88	88	88	88	88		85	85	85	85	85	
Cost of sensitization (B)=(B1 × B2)+(B3 × B4)	23,671	23,671	23,671	23,671	23,671	118,356	20,726	20,726	20,726	20,726	20,726	103,631
**III. Coordination (incl. supervision and M&E)**												
Annual cost of coordination (central level) (C1)	11,106	11,106	11,106	11,106	11,106		41,420	41,420	41,420	41,420	41,420	
Annual cost of coordination per region (C2)	14,357	14,357	14,357	14,357	14,357		30,999	30,999	30,999	30,999	30,999	
Cost of coordination (C)=(C1)+(B1 × C2)	370,028	370,028	370,028	370,028	370,028	1,850,141	351,407	351,407	351,407	351,407	351,407	1,757,036
**IV. Training cost**												
Number of school (primary &secondary) (D1)	8,537	8,612	8,688	8,765	8,842	43,444	26,214	26,680	27,155	27,639	28,131	135,819
Teachers to train per school (D2)	2						2					
Number of teachers to train (D3) = (D1) × (D2)	17,075	–	17,376	–	17,684	52,135	52,428	–	54,311	–	56,261	163,000
Ratio trainer/teacher (D4)	18						18					
Number of trainers to train (D5)=(D3) * (D4)	98					98	300					300
Cost per teacher to train (D6)	100						78					
Cost per trainer to train (D7)	472						495					
Training cost: (D)=(D3 × D6)+(D5 × D7)	1,754,615	–	1,738,721	–	1,769,457	5,262,792	4,234,674	–	4,233,067	–	4,385,105	12,852,847
**V. Screening by teachers**												
Screening kit costs per school (E1)	4						4					
Schools requiring screening kits (E2)	8,537	75	76	76	77	8,842	26,214	467	475	483	492	28,131
Cost for screening (E)=(E1) × (E2)	35,479	312	315	318	321	36,744	113,103	2,013	2,049	2,086	2,123	121,373
**VI. Treatment by mobile refraction team**												
Nb of schools covered by mobile team/day (F1)	4						4					
Cost per mobile refraction team/day (F2)	340	340	340	340	340		275	275	275	275	275	
Cost for mobile team (F)=(D1/F1) × (F2)	726,284	732,675	739,123	745,627	752,188	3,695,897	1,802,541	1,834,627	1,867,283	1,900,521	1,934,350	9,339,323
**VII. Provision of spectacles**												
Number of school children to be screened (G1)	2,647,911	650,432	994,883	599,823	584,565	5,477,614	4,429,018	969,388	1,849,051	935,846	914,888	9,098,192
% children requiring ready-made spectacles (G2)	0.40%	0.40%	0.40%	0.40%	0.40%		0.54%	0.54%	0.54%	0.54%	0.54%	
Ready-made spectacles for children(G3)=(G1xG2)	10,643	2,614	3,999	2,411	2,350	22,016	24,121	5,279	10,070	5,097	4,983	49,550
Average number of teachers per school (G4)	7						8					
Nb of teachers to be screened (G5)=(E2xG4)	62,483	550	555	560	564	64,712	206,394	3,674	3,739	3,806	3,874	221,486
% teachers requiring near-vision spectacles (G6)	60%						60%					
Ready-made spectacles for teachers												
(G7)=(G5xG6)	37,490	330	333	336	339	38,827	123,836	2,204	2,244	2,283	2,324	132,892
Total number of ready-made spectacles required (G8)= (G3) + (G7)	48,133	2,944	4,332	2,747	2,688	60,843	147,957	7,484	12,314	7,380	7,307	182,441
Price for ready-made spectacles (G9)	2	2	2	2	2		2	2	2	2	2	
Total cost of already made spectacles; (G10)=(G8)x(G9)	103,837	6,352	9,344	5,925	5,799	131,257	319,188	16,145	26,564	15,921	15,763	393,581
% custom-made spectacles for children (G10)	0.18%	0.18%	0.18%	0.18%	0.18%		0.23%	0.23%	0.23%	0.23%	0.23%	
Nb of custom-made spectacles required (G11)=(G1) × (G10)	4,683	1,150	1,759	1,061	1,034	9,687	10,087	2,208	4,211	2,131	2,084	20,721
Price for custom-made spectacles (G12)	7	7	7	7	7		27	27	27	27	27	
Total cost of customized spectacles; (G13)=(G11) × (G12)	32,373	7,952	12,163	7,333	7,147	66,969	269,574	59,002	112,543	56,961	55,685	553,765
Total cost of spectacles (G)=(G10) + (G13)	136,210	14,304	21,508	13,259	12,946	198,226	588,762	75,147	139,107	72,882	71,448	947,346
**VIII. Referral to eye clinic**												
Number of children referred to eye clinics (H1)	4,683	1,150	1,759	1,061	1,034	9,687	32,453	7,103	13,549	6,857	6,704	66,667
Transportation allowance per child (H2)	22	22	22	22	22		24	24	24	24	24	
Total cost for referrals (H)=(H1) × (H2)	101,022	24,815	37,956	22,884	22,302	208,980	784,059	171,609	327,333	165,671	161,961	1,610,632
**Total cost =** (A)+(B)+(C)+(D)+(E)+(F)+(G)+(H)	3,151,219	1,165,805	2,931,322	1,175,787	2,950,913	11,375,046	7,918,792	2,455,529	6,940,973	2,513,292	6,927,120	26,755,707

The first year is the most expensive for both countries due to programme start-up expenditures, capacity building and training activities, as well as the requirement of the SHIP guidelines to screen all children in primary and lower secondary schools in year one. In subsequent years, the guidelines recommend screening new entrants, children in secondary school (every other year) and re-examine children who were given spectacles previously. As a result, the programme start-up costs required for scale up were estimated at $1,758,525 in Cambodia and $4,255,400 in Ghana. Cost of screening and refractive error correction in year one was estimated at $1,392,694 in Cambodia and $3,660,599 in Ghana. With a training session every two years, recurrent expenditures in the subsequent year in Cambodia amounted to $1,165,805 in year two and $2,455,529 in Ghana.

Table 3 indicates the cost and output breakdown per province/district for a five-year programme. It is anticipated that scaling up the school-based vision screening programme based on SHIP guidelines will lead to 5.5 million children being screened in Cambodia, with 31,703 receiving spectacles and another 9,687 being referred for further examination or treatment. In Ghana, over 9 million school aged children will be screened, among whom 70,270 will receive spectacles and 66,667 referred to an eye care facility for further examination or treatment.

**Table 3:** Cost and outputs of national school-based vision screening programmes in Ghana and Cambodia, projections by district/ province for 2019–2023 (in USD 2020).

Country	Administrative unit	Total cost (USD)	Teachers trained	Schools covered	Children screened	Children examined	Spectacles provided	Children referrals
Cambodia	Banteay Meanchey	596,999	944	2,403	258,833	10,643	1,498	458
	Battambang	890,837	1,474	3,749	432,211	8,644	2,502	764
	Kampong Cham	631,822	995	2,532	382,873	7,657	2,216	677
	Kampong Chhnang	451,682	678	1,725	206,387	4,128	1,195	365
	Kampong Speu	511,644	780	1,983	305,427	6,109	1,768	540
	Kampong Thom	684,559	1,109	2,822	264,560	5,291	1,531	468
	Kampot	507,717	782	1,989	220,597	4,412	1,277	390
	Kandal	621,011	969	2,465	430,574	8,611	2,492	761
	Kep	105,394	53	135	13,690	274	79	24
	Koh Kong	229,567	279	709	44,698	894	259	79
	Kratie	425,166	635	1,616	157,192	3,144	910	278
	Mondul Kiri	187,197	202	513	38,596	772	223	68
	Otdar Meanchey	342,159	486	1,238	96,923	1,938	561	171
	Pailin	137,630	112	285	28,079	562	163	50
	Phnom Penh	313,328	395	1,005	381,071	7,621	2,206	674
	Preah Sihanouk	180,093	185	471	72,408	1,448	419	128
	Preah Vihear	373,369	543	1,383	97,308	1,946	563	172
	Prey Veng	793,106	1,292	3,288	415,838	8,317	2,407	735
	Pursat	462,391	702	1,787	182,772	3,655	1,058	323
	Ratanak Kiri	321,282	444	1,129	98,329	1,967	569	174
	Siemreap	743,998	1,199	3,050	434,787	8,696	2,516	769
	Stung Treng	258,767	334	849	59,303	1,186	343	105
	Svay Rieng	433,078	643	1,636	202,915	4,058	1,174	359
	Takeo	588,761	918	2,336	350,115	7,002	2,026	619
	Tbaung Khmum	586,766	922	2,346	302,127	6,043	1,749	534
	All provinces (n = 25)	11,378,325	17,075	43,444	5,477,614	115,018	31,703	9,687
Ghana	Ashanti	4,369,615	8,774	22,730	1,576,835	31,537	12,179	11,554
	Brong Ahafo	3,121,079	6,195	16,048	1,036,424	20,728	8,005	7,594
	Central	2,774,927	5,546	14,368	781,537	15,631	6,036	5,727
	Eastern	3,195,649	6,452	16,714	893,214	17,864	6,899	6,545
	Greater Accra	1,808,409	3,356	8,695	698,234	13,965	5,393	5,116
	Northern	3,187,660	6,236	16,156	1,233,284	24,666	9,525	9,037
	Upper East	1,427,443	2,565	6,645	543,930	10,879	4,201	3,986
	Upper West	1,276,391	2,262	5,861	464,586	9,292	3,588	3,404
	Volta	2,780,618	5,470	14,170	928,186	18,564	7,169	6,801
	Western	2,826,544	5,571	14,433	941,962	18,839	7,275	6,902
	All regions (n = 10)	26,768,335	52,428	135,819	9,098,192	181,964	70,270	66,667

### 3.3 Sensitivity analysis

**Fig. 2:**
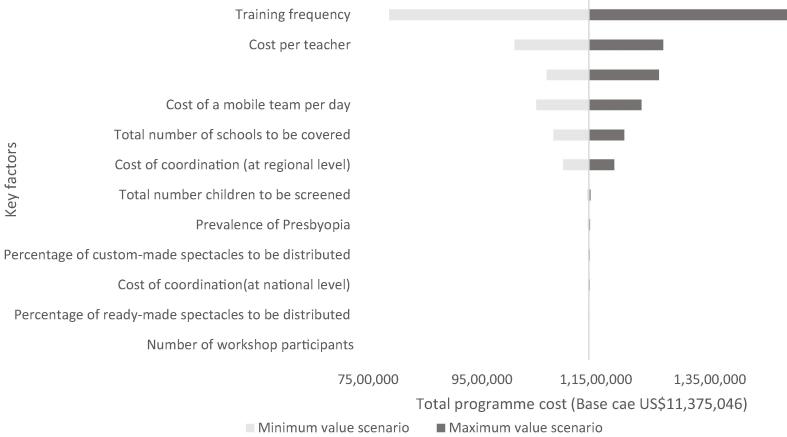
Univariate sensitivity analysis for school-health integrated programming in Cambodia (in USD 2020).

**Fig. 3:**
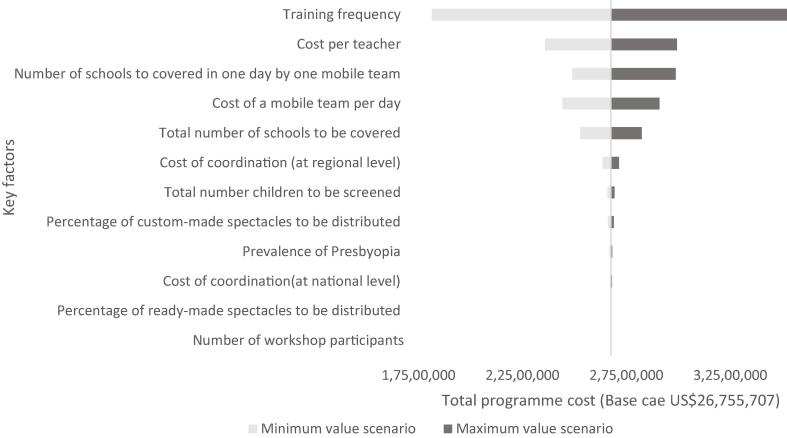
Univariate sensitivity analysis for school-health integrated programming in Ghana (in USD 2020).

Fig. 2, Fig. 3 show results of the univariate sensitivity analysis. Six model parameters significantly impact the budget when their base case value is increased or decreased by 25%, or changing training frequency from annual, every two years or every five years. These parameters are the same for Cambodia and Ghana, although the order of importance of these parameters varies slightly.

In Cambodia, changes in the training frequency, the cost per teacher trained, the number of schools covered by the mobile team each day, and the cost of the mobile refraction team per day produce the largest impacts on the five-year base case budget estimate ($7,866,869-$14,852,621). This is followed by the total number of schools to be covered and the cost of coordination (at regional level) which influence the total programme cost (Fig. 2).

In Ghana, changes in the training frequency, the cost per teacher, the number of schools covered by the mobile team per day, and the cost of the mobile team per day have a large impact on the budget estimate ($18,137,534-$35,223,159). This is followed by the total number of schools covered, and the cost of coordination per region (Fig. 3).

Based on the multivariate sensitivity analysis, the projected total cost over five years ranges from $5,961,380 to $19,490,120 in Cambodia (base case value: $11,375,046), and from $13,399,511 to $46,705,375 in Ghana (base case $26,746,857) (Table 4 and Table 5). This means that in both countries, the total cost of the five-year programme could be about 50% lower than the estimated base case value in the best-case scenario and 75% higher in the worst-case scenario.

**Table 4:** Results of multivariate analysis for Cambodia, total cost of school-health integrated programming for different scenarii(USD 2020).

CAMBODIA	Training frequency	Min (Every five years)			Base case (Every two years)			Max (Annual)		
Cost of mobile team per day	Number of schools to cover in one day by one mobile team Cost per teachers	Max (5)	Base case (4)	Min (3)	Max (5)	Base case (4)	Min (3)	Max (5)	Base case (4)	Min (3)
Min ($255)	Min ($75)	5,961,380	6,515,765	7,439,739	8,592,513	9,146,898	10,070,872	11,200,694	11,755,079	12,679,053
	Base case ($100)	6,388,510	6,942,894	7,866,869	9,896,687	10,451,072	11,375,046	13,374,262	13,928,647	14,852,621
	Max ($125)	6,815,639	7,370,024	8,293,998	11,200,861	11,755,246	12,679,220	15,547,830	16,102,214	17,026,188
Base case ($340)	Min ($75)	6,700,560	7,439,739	8,671,705	9,331,693	10,070,872	11,302,838	11,939,874	12,679,053	13,911,019
	Base case ($100)	7,127,689	7,866,869	9,098,834	10,635,867	11,375,046	12,607,012	14,113,441	14,852,621	16,084,587
	Max ($125)	7,554,819	8,293,998	9,525,964	11,940,041	12,679,220	13,911,186	16,287,009	17,026,188	18,258,154
Max ($425)	Min ($75)	7,439,739	8,363,713	9,903,670	10,070,872	10,994,846	12,534,804	12,679,053	13,603,027	15,142,985
	Base case ($100)	7,866,869	8,790,843	10,330,800	11,375,046	12,299,020	13,838,977	14,852,621	15,776,595	17,316,552
	Max ($125)	8,293,998	9,217,973	10,757,930	12,679,220	13,603,194	15,143,151	17,026,188	17,950,163	19,490,120

**Table 5:** Results of multivariate analysis for Ghana, total cost of school-health integrated programming for different scenarii (USD 2020).

Ghana	Training frequency	Min (Every five years)			Base case (Every two years)			Max (Annual)		
Cost of mobile team per day	Number of schools to cover in one day by one mobile team Cost per teachers	Max (5)	Base case (4)	Max (3)	Max (5)	Base case (4)	Max (3)	Max (5)	Base case (4)	Max (3)
Min ($255)	Min ($75)	13,399,511	14,772,278	17,078,978	19,863,140	21,235,908	23,542,608	26,213,729	27,586,497	29,893,197
	Base case ($100)	14,421,086	15,793,853	18,100,553	23,039,258	24,412,026	26,718,726	31,506,710	32,879,478	35,186,178
	Max ($125)	15,442,661	16,815,428	19,122,128	26,215,376	27,588,144	29,894,844	36,799,691	38,172,459	40,479,159
Base case ($340)	Min ($75)	15,267,375	17,107,109	20,192,086	21,731,005	23,570,739	26,655,716	28,081,594	29,921,328	33,006,304
	Base case ($100)	16,288,950	18,128,684	21,213,661	24,907,123	26,746,857	29,831,834	33,374,575	35,214,309	38,299,286
	Max ($125)	17,310,525	19,150,259	22,235,236	28,083,241	29,922,975	33,007,952	38,667,556	40,507,290	43,592,267
Max ($425)	Min ($75)	17,135,240	19,441,940	23,305,194	23,598,869	25,905,569	29,768,823	29,949,458	32,256,158	36,119,412
	Base case ($100)	18,156,815	20,463,515	24,326,769	26,774,987	29,081,688	32,944,941	35,242,439	37,549,139	41,412,393
	Max ($125)	19,178,390	21,485,090	25,348,344	29,951,106	32,257,806	36,121,060	40,535,421	42,842,121	46,705,375

## 4 Discussion

Prior economic analyses of school-based vision screening programmes in LMICs rely primarily on information from secondary sources or WHO-CHOICE standardized unit costs. This is one of the first studies to estimate costs using real-world data from two countries, Cambodia and Ghana. This study also carried out a budget impact analysis to assess the affordability of delivering these interventions at scale in the studied settings.

The share of resources required for the actual screening and refractive error correction were relatively modest, supporting the conclusions of earlier research that vision screening for schoolchildren by teachers is effective and less costly than alternative primary eye care models [38].

In this study, we did not have access to students’ personal records on disability or special needs status. We are not aware of any reported cases where the standard screening procedure was not applicable to certain children. However, we acknowledge that it may be the case for children with some severe disabilities. We suggest that children unable to be tested using the screening protocol should be referred to optometrists who are better suited to examine complex cases, including children with special needs. Alternative ways of screening are also available and could eventually be added. For example Gogate et al., in India, used Kay pictures as an alternative to Snellen’s tumbling E chart for young children and children with severe learning disabilities [39].

The standard unit cost based on the SHIP technical guidelines make cross-country estimates more comparable and highlight a number of context-specific differences which need to be taken into account when planning similar interventions in other settings. Except for the cost of the mobile team, the unit costs for all activities were higher in Ghana. These differences are likely to be due to different salary levels, per diem rates and accommodation, and rental costs. The results show the importance of using context-specific data when planning and budgeting interventions at scale.

It is difficult to place our estimates and projections in the context of other studies, as evidence on cost of school-based vision screening is scarce and the differences in methodological approaches of the few studies available limit opportunities for meaningful comparisons. In addition, this analysis did not aim to calculate the number of DALYs averted by correction of refractive error. Therefore, at this stage it is not possible to do a comparison with the WHO cost-effectiveness thresholds [40] or cost per DALY estimates presented in earlier studies [17], [18]. The only estimates with which we can compare our results are from Baltussen, Naus & Limburg (2009), who reported the cost per child treated for refractive error. However, their estimates were made for children aged 11 + years and are available for the WHO Africa region only. They find that the cost per child treated for refractive error is I$204, when screening children aged 11–15 years and I$450, when screening 13-year-olds only [17]. When converting our results into the same currency (international dollars with the same base year), we obtain estimates of I$802 for Cambodia and I$726 for Ghana (dividing the projected number of children provided with spectacles by the total programme cost).

Our analysis also highlighted several programmatic aspects of vision screening which should be considered when designing and implementing similar programmes as they could potentially help reduce costs and enhance the benefits of school-based programmes. Firstly, most children with refractive error identified by the SHIP project were eligible for ready-made spectacles. Considering the price difference between ready-made and custom-made spectacles ($2 vs $6.5 in Cambodia and $2 vs $25 in Ghana), the use of ready-made spectacles provided on site should be prioritised, as it can generate substantial savings. Secondly, the use of teachers for conducting the initial vision screening is essential, as it significantly reduces the cost of the overall programme. Thirdly, the costs of activities and inputs shown to be the key cost drivers, such as stakeholder mobilisation or teacher training, can be minimised if these activities are delivered as part of other planned programmes or training activities. Fourthly, as a large proportion of the costs required at the start of the vision screening programme are fixed, the delivery of the programme at scale would maximise the number of beneficiaries and reduce the unit cost per output achieved over the life of the programme. Finally, considering that teacher training and coordination constituted a significant part of the overall budget of the programmes at scale, we recommend further integration of vision screening with other school-based interventions to generate economies of scope. For example, training of teachers could cover other essential interventions, such as hearing screening, deworming, and health education [16].

One of the main purposes of conducting a budget impact analysis is to assess the affordability of a new intervention in a specific setting. The total budgets required to implement the national school-based vision screening programmes in Cambodia and Ghana were $11,375,046 and $26,755,707 over five years (2019–2023) respectively. It is therefore important to put the projected budget estimates in perspective with the education sector expenditure in each country.

In Cambodia, the national education budget in 2018 was equivalent to $848 million (Nary, 2017; Sokhean, 2017; Sophirom, 2017). In Ghana, it was $2.074 billion [41]. The delivery of the school-based vision screening programmes at scale is judged to be affordable in both countries, as the estimated five-year budget required for school-based vision screening would represent only 1.34%, 0.70% in the best-case scenario or 2.30% in the worst-case scenario, of the current annual education budget in Cambodia. In Ghana, it would represent 1.29%, 0.65% in the best-case scenario or 2.25% in the worst-case scenario of the annual budget. If we assume that the annual education budget remains constant during the 5-year period in both countries, the overall budget for the programme would represent only 0.27% of the five-year education budget in Cambodia and 0.26% in Ghana.

This study has several limitations which need to be considered when interpreting and using these findings. Firstly, the budget impact analysis is based on an important assumption that eye care services and skilled eye health personnel are available and sufficient for implementing the programme nationally. This assumption may not hold for all provinces or regions of the two countries. If the scale up of the vision screening programme requires investments in new infrastructure and human resources, the budget implications will be considerable. Secondly, the impact of COVID-19 has not been explored in our projections. The SHIP methodology is currently being adapted to embed the necessary social distancing measures, personnel protective equipment for optometrists, and adapted training sessions. This will have a financial impact on the implementation of school-based vision screening that has not been assessed yet. Moreover, national education budgets might be affected by the consequent economic recession in Cambodia and Ghana [42], [43]. Thirdly, there are limitations regarding the availability of data used in the projection model including the prevalence of visual impairment and refractive error in school-age children. Education data was also unavailable for some administrative regions and the national averages were applied in the model. Moreover, data on sensitivity and specificity of teacher’s screening ability, following the SHIP training, are still missing. The current literature show evidence supporting the approach of using teachers as first screener, but other examples show over-referrals, or even in some case under referrals, depending on the level of training of teachers. Fourthly, our analysis estimates the budget impact of integrating vision screening in government schools only and does not account for children enrolled in private schools or out-of-school. We did not have access to information on private schools in the countries. As private schools tend to cater for the needs of wealthier social groups, children from such families are more likely to access RE services through the private sector and it is unlikely that the governments in low-income settings would be providing these services. However, the calculations for budget impact analysis suggested in this paper can also be used by the private sector or private insurance schemes, should such services be provided at scale by the private sector education providers. Finally, this study estimated the incremental cost of integrating vision screening into the existing SHN programmes and should not be interpreted as the true economic cost of the intervention.

## 5 Conclusion

This study suggests that the scale up of school-based vision screening programmes in resource limited settings, such as Cambodia and Ghana, is affordable for the current education budgets, providing there is sufficient in-country capacity to deliver such interventions at scale. The study highlights several policy and programme implications and provides suggestions for minimising costs and maximising efficiencies of vision screening in a school setting. Findings from this analysis can help education planners and international partners to improve their planning and budgeting processes for school-based interventions to improve health and learning outcomes for children in low- and middle-income countries.

## Funding

This work was supported by the World Bank and Global Partnership for Education [contract number 8005499].

## CRediT authorship contribution statement

**Thomas Engels:** Conceptualization, Methodology, Validation, Formal analysis, Investigation, Resources, Data curation, Writing - original draft, Writing - review & editing, Supervision, Project administration. **Guillaume Trotignon:** Conceptualization, Methodology, Software, Validation, Formal analysis, Investigation, Resources, Data curation, Writing - original draft, Writing - review & editing, Supervision, Project administration, Visualization. **David Agyemang:** Resources, Investigation, Writing - review & editing. **Imran Khan:** Conceptualization, Resources, Funding acquisition, Supervision, Writing - review & editing. **Kann Puthy:** Resources, Writing - review & editing. **Liesbeth Roolvink:** Resources, Investigation, Writing - review & editing. **Elena Schmidt:** Supervision, Writing - review & editing.

## Declaration of Competing Interest

Thomas Engels, Guillaume Trotignon, David Agyemang, Imran Khan, Kann Puthy, Liesbeth Roolvink, and Elena Schmidt declare not conflict of interest.

## Figures and Tables

**Table A1:** Summary of budget impact analysis included activities.

Implementation financial costs of screening of school children
**I. Capacity and partnership strengthening (workshop)**
**Number of participants (A1)**
**Cost per participants (A2)**
**Cost of capacity building, collaboration workshop (A)=(A1) × (A2)**
**II. Community sensitisation**
**Number of region (B1)**
**Cost per region (B2)**
**Number of district (B3)**
**Cost per district (B4)**
**Total cost of community sensitization (B)=((B1) × (B2)) + ((B3) × (B4))**
**III. Coordination (incl. supervision and M&E)**
**Cost of coordination per year (at national level) (C1)**
**Cost of coordination per year per region (C2)**
**Total Cost of coordination and supervision (C)=(C1) + ((B1) × (C2))**
**II. Training cost**
**Total number of school (primary and secondary) (D1)**
**Teachers to train per number of school (D2)**
**Number of teachers to train (D3) = (D1) × (D2)**
**Trainers to train per teachers, 2 trainers for 35 teachers (D4)**
**Number of trainers to train, 10 training session per trainer (D5)=((D3)/(D4))/10**
**Cost per teacher to train (D7)**
**Cost per trainer to train (D6)**
**Training cost (Year one only) (D)=(D3) × (D7) + (D5) × (D6)**
**III. Screening at schools**
**Screening kit costs per school (cards, ropes etc.) (E1)**
**Number of schools (E2)**
**Total cost for material (E)=(E1) × (E2)**
**IV. Treatment by mobile team**
**Number of schools covered by optometrist mobile team per day (F1)**
**Cost per day per optometrist mobile team (F2)**
**Total by optometrist (F)= ((D1) / (F1)) × (F2)**
**VI. Cost of spectacles**
**Total number of school children to be screened per year (G1)**
**Proportion of children requiring ready-made spectacles (G2)**
**Total number of children requiring ready-made spectacles (G3)=(G1) × (G2)**
**Ratio teacher per school (G4)**
**Number of teachers to screen (G5) = (E2) × (G4)**
**Proportion of teacher requiring ready-made spectacles (G6)**
**Total number of teachers requiring ready-made spectacles (G7)=(G5) × (G6)**
**Number of already made spectacles provided (G8)=(G3) + (G7)**
**Price of ready-made spectacles (G9)**
**Total cost of already made spectacles; (G10)=(G8)x(G9)**
**Proportion of children requiring custom-made spectacles (G10)**
**Number of custom-made spectacles provided (G11)=(G1) × (G10)**
**Price of custom-made spectacles (G12)**
**Total cost of customized spectacles; (G13)=(G11) × (G12)**
**Total cost of spectacles (G)=(G10) + (G13)**
**VII. Referral to eye clinic**
**Number of children referred to eye clinic (by teachers and optometrist) (H1)**
**Transportation cost per child (H2)**
**Total cost transportation for referred children (H)=(H1) × (H2)**
Total incremental cost = (A) + (B) + (C) + (D) + (E) + (F) + (G) + (H)

**Table B1:** School-health integrated programming cost projection model: inputs, assumptions, and sources.

Parameter	Cambodia	Ghana	Data source
Administrative units			
Number of provinces/regions	25	10	MLGRD (2006), Statistics Bureau (2013)
Number of districts	196	210	
Education sector data			
Enrolment in primary school (2016/2017)	2,022,061	3,258,996	MOEYS (2017), MOE (2017)
Enrolment in lower secondary school (2016/2017)	586,042	1,256,908	
Number of schools (primary and lower secondary) 2016/2017	8,389	25,305	
Number of teachers per school (primary and lower secondary) 2016/2017	7	8	
Growth rate for new entrants in primary schools	−1.08%	−1.33%	Authors‘ projection based on data from MOEYS (2017), MOE (2017), and FHI360 (2012)
Growth rate for number of schools	0.88%	1.78%	
Capacity building			
Number of participants for national capacity building and partnership strengthening workshop	48	55	Authors’ calculation based on project data; assuming one coordinator per administrative unit (region or province)
Number of teacher to train per school	2	2	SHIP guidelines (2016) and project data
Trainers to train per teachers	17.5	17.5	Authors’ calculation based on SHIP guidelines (2016) and assuming a ratio of two trainers for 35 teachers
Number of training of teachers session per trainer	10	10	
Number of trainers to be trained	98	300	
Training frequency	Every 5yrs	Every 5yrs	Baltussen, Naus & Limburg (2009)
Screening by teachers			
Screening material costs (tape, card etc.)	$3	$4	Project data and experts opinion
Useful life for screening kit	5 years	5 years	
Number of screening kit per school	1	1	
Eye examination by mobile refraction team:			
Proportion of children referred by teachers	2%	2%	Project data and experts opinion
Number of schools visited by Mobile refraction team per day	4	4	
Prevalence of Presbyopia among school teachers	60%	60%	Based on experts opinion and data presented in Holden et al. (2008) and Bourne et al. (2017)
Proportion of children provided with ready-made spectacles	0.4%	0.54%	Project data
Proportion of children provided with custom-made spectacles	0.18%	0.23%	
Proportion of children referred to health clinics	0.18%	0.73%	

**Table C1:** School-health integrated programming budget impact analysis target population for Cambodia and Ghana.

Target population	Cambodia	Ghana	Data source
*Teachers*			
Year 1: All teachers in primary and lower secondary	62,483	206,394	Number of teachers projected by authors using flow model and data from MOEYS (2017) and MOE (2017)
Year 2: New teachers only	550	3674	
Year 3: As per year 2	555	3739	
Year 4: As per year 2	560	3806	
Year 5: As per year 2	564	3874	
*Students*			
Year 1: All children in primary and lower secondary	2,647,911	4,429,018	SHIP guidelines (2016); number of school children projected by authors using flow model and data from MOEYS (2017) and MOE (2017)
Year 2: New entrants in primary and lower secondary, and children given spectacles in previous year	665,758	1,027,717	
Year 3: New entrants in primary school, all children in lower secondary, and children given spectacles in previous year	998,737	1,862,585	
Year 4: As per year 2	605,604	960,376	
Year 5: As per year 2	588,070	927,536	

## Appendix A

See Table A1, Table B1, Table C1
